# A Cumulative Effect of Food and Viruses to Trigger Celiac Disease (CD): A Commentary on the Recent Literature

**DOI:** 10.3390/ijms22042027

**Published:** 2021-02-18

**Authors:** Maria Vittoria Barone, Salvatore Auricchio

**Affiliations:** 1 Department of Translation Medical Science (DISMET), University Federico II, 80131 Naples, Italy; 2 European Laboratory for the Investigation of Food Induced Diseases (ELFID), University Federico II, 80131 Naples, Italy; salauric@unina.it

**Keywords:** celiac disease, gliadin, inflammation, Mediterranean diet, virus, inflammatory chronic disease

## Abstract

Celiac disease (CD) is a type of inflammatory chronic disease caused by nutrients such as gliadin that induce a TC (T cell)-mediated response in a partially known genetical background in an environment predisposed to inflammation, including viruses and food. Various experimental and clinical observations suggest that multiple agents such as viruses and bacteria have some common, inflammatory pathways predisposing individuals to chronic inflammatory diseases including celiac disease (CD). More recently, a Western diet and lifestyle have been linked to tissue inflammation and increase in chronic inflammatory diseases. In CD, the gliadin protein itself has been shown to be able to induce inflammation. A cooperation between viruses and gliadin is present in vitro and in vivo with common mechanisms to induce inflammation. Nutrients could have also a protective effect on CD, and in fact the anti-inflammatory Mediterranean diet has a protective effect on the development of CD in children. The possible impact of these observations on clinical practice is discussed.

## 1. Introduction

Autoimmune diseases affect about 5% of the population [1]. They are result of interactions between genetic susceptibility, often related to human leukocyte antigen (HLA) class I and II genes, and environmental factors. An important role in autoimmunity is the association between infectious agents and autoimmune diseases. In fact, the onset of various autoimmune diseases including rheumatoid arthritis, type 1 diabetes, and hepatitis has been reported to be in association with a variety of infections [2,3,4,5]. Although the association between autoimmune disease and infections has been proven in many cases, only in a few of them has the link with a specific infection been identified as the cause of the disease. One example is rheumatic heart disease, which can follow infection with group A streptococci, caused by molecular mimicry between cardiac and streptococcal antigens [6].

Celiac disease (CD) is a type of inflammatory chronic disease induced by a food protein such as gliadin with a T cell (TC)-mediated mechanism [7] that develops in genetically predisposed individuals in an environment predisposed to inflammatory reactions, including viruses and food (in particular gliadin) [8].

The objective of this review is to summarize the recent literature on the possible combined role of viruses and food as triggers for CD.

## 2. Results

### 2.1. Role of Infections in CD

The link between infections and CD has been established clinically by several large-scale, population-based cohort studies. The Teddy study [9] and the Norwegian Mother and Child Cohort Study [10] on 6327 and 72,921 children, respectively, showed that early life infections may play a role in CD development and that rotavirus vaccination reduces the risk of CD [9,11]. Other studies also confirmed the role of infections and in particular enterovirus and rotavirus infections in the onset of CD in populations at risk [12,13]. Moreover, CD patients have a higher titer of antibodies against reovirus or human adenovirus serotype 2 [14,15]. Norovirus and reovirus infections have the capacity to interfere with the development of tolerance to an oral antigen in mice HLA DQ8 [16,17].

The role of bacterial infection is still debated in CD. Azimi et al. published a comprehensive review regarding the role of bacterial infections, both pathogenic and non-pathogenic, in CD [18]. In particular, Campylobacter infections [19] seem to have a role in the pathogenesis of CD whereas Helicobacter can be excluded [20]. Interestingly, T cell receptor cross-reactivity between gliadin and bacterial peptides has been described in CD, indicating that molecule mimicking can be one of the disease mechanisms [21,22].

Taken all together, these clinical and experimental observations suggest that multiple agents such as viruses and bacteria have some common, inflammatory pathways predisposing humans to CD.

### 2.2. Nutrients Are Able to Induce and Regulate Tissue Inflammation

There is increasing evidence that the diet can modulate tissue inflammation and therefore the onset of several different disorders. Nutrients and inflammation are strictly linked, and in fact carbohydrates, lipids, salt and, in general, Western diet and lifestyle, have been linked to tissue inflammation [23]. Western diets (poor in polyphenol products and enriched in fat, phosphatidylcholine, L-carnitine, sugars, and other pro-inflammatory food components) promote inflammation. Numerous studies have also demonstrated the negative effects of this diet on gut microbiota richness and function. It is now generally acknowledged that nutrients can shape the composition of the microbiota and that the Western-style diet, causing disorders of the microbiota, intensifies the chronic inflammatory process, and consequently leads to the development of chronic inflammation. Gut microbiota dysbiosis is involved in the pathogenesis of diverse diseases, such as metabolic syndrome, cardiovascular diseases, celiac disease, inflammatory bowel disease, neurological disorders, and ageing. [23,24].

On the other side, the Mediterranean diet (MD) has anti-inflammatory effects that now have been well studied [22,23]. Adherence to a MD has been linked to a reduced incidence of obesity and metabolic syndrome and decreased mortality and morbidity in patients with cardiovascular diseases [23,24]. The traditional MD is characterized by a high content of polyphenol-rich products (extra-virgin olive oil and red wine) vegetables, grains, legumes, wholegrain cereals, nuts, a beneficial proportion of fatty acids (high in monounsaturated fatty acids and polyunsaturated fatty acids, low in saturated fatty acids), and low consumption of processed meat and refined sugars. Growing experimental and clinical evidence also suggests that a MD contributes to a beneficial gut microbiota pattern. Several previous studies have described how a MD could positively affect gut microbial communities. This diet pattern, in fact, positively affects the diversity and activity of various gut bacteria and hence improves host metabolism [24]. In conclusion, dietary patterns can affect inflammation and disease in several different ways.

### 2.3. Gliadin and Inflammation in CD

Celiac disease (CD) is an inflammatory chronic disease induced by food. Gliadins, important storage proteins in wheat, are the main factors that induce CD in genetically susceptible individuals. They are difficult to digest proteins. Several studies, in fact, using in vitro multi-compartment models that include all the phases of digestion, demonstrated that the A-gliadin recombinant protein was largely digested except for two main peptides, 25-mer (P31–55, containing P31–43) and 33-mer (P57–89) [25,26,27]. These data indicate that certain gliadin peptides such as P31–43 are particularly resistant to digestion by gastric, pancreatic, and brush border membrane enzymes, and therefore, they can come into contact with the intestinal epithelium in our everyday life. In fact, these peptides survive intestinal digestion in vivo [28] and are able to induce different effects in CD cells and tissues.

The best studied peptide is 33-mer, which is presented by antigen-presenting cells to TC, leading to activation of adaptive immunity [7]. On the other side, P31–43 is not presented to TC [29] but is able to induce several different effects in cells and mice including innate immune response activating the interferon (INF) alpha pathway [30,31] via Toll-like receptor (TLR) signaling [30]. Moreover, in cultured intestines of subjects with CD, but not in controls, P31–43 activates INF, a key mediator of the immune response in CD [30], indicating a specificity of effects on CD intestine.

Gliadin and the undigested gliadin peptide p. 31–43 have also been shown to be able to induce inflammation in CD. In particular this peptide and the peptic tryptic product of gliadin can induce NFkB and the other markers of inflammation in vitro and in vivo [25,32,33].

One of the mechanisms by which P31–43 can activate inflammation is due to its effects on vesicular trafficking [25]. This peptide shares a sequence homology with HRS (hepatocyte growth factor regulated tyrosine kinase), the major regulator of endocytic vesicle maturation from early to late vesicles. Silencing or mutation of this protein induces delay of vesicular trafficking and activation of several different pathways due to the delay of decay of the receptors present on the early vesicles. P31–43, due to its sequence similarity with HRS, competes with its correct localization to the early endocytic compartment, delaying the endocytic trafficking in the same way as HRS silencing or mutation. All this results in inducing the same biological effects of HRS silencing or mutation including the activation of the inflammatory pathways. In Table 1 we show a comparison between HRS silencing and several stressors such as P31–43 and viral ligand (loxorubine Poly I:C) effects on different pathways (Table 1). Interestingly, a delay of the endocytic vesicles and the activation of the inflammatory pathway is also present in the enterocytes of CD subjects independently of gliadin presence [25].

### 2.4. Cooperative Mechanisms between Gliadin and Infections Inducing Tissue Inflammation

The intestinal cells, particularly enterocytes and dendritic cells, can sense nutrients and respond by activating pro-inflammatory or anti-inflammatory mediators partially through the same mechanisms which recognize viruses or bacteria (i.e., TLRs). An example of nutrients that can interact with TLRs are the ATI (amylase trypsin inhibitor) non gluten proteins, which are very resistant to intestinal proteases contained in wheat which can initiate innate immune activation through TLR-4 signaling [34]. These proteins in wild mice can induce inflammation and increase intestinal permeability but cannot induce mucosal damage. Moreover, in non-obese diabetic (NOD) HLA DQ8 mice, ATIs can have an adjuvant role to the intestinal damage induced by gluten [35].

Higher gluten intake has been proposed as an environmental trigger for CD. Though, more recently some papers indicate that the amount of the gluten in the diet plays only a minor role in the incidence of CD [36].

On the other side, a study on CD patients at genetic risk of the disease has both confirmed that enterovirus infections are associated with increased risk and highlighted that further increased risk is conferred by interaction between enterovirus and higher gluten intake in the diet. All this indicates cumulative effects between frequently used nutrients such as gliadin and viral infections [37]. In the recent literature there are some reports that could give indications on the mechanisms of this cumulative effect. Silencing of HRS, P31–43, and viral ligands such as loxorubine, Poly I:C and ATIs share common pathways in vitro and in vivo (Table 1).

P31–43 reinforces the INF alpha-mediated immune response to viruses in enterocytes. In fact, there is a cooperation between this peptide and loxorubine, a TLR7 ligand, to activate the downstream signaling [30]. P31–43 activated IFN-α, a mediator of the innate immune response in CD, in the intestine of subjects with CD and in an enterocyte cell line, CaCo-2. P31–43 cooperated with a viral ligand to activate the TLR7 pathway by interfering with endocytic trafficking [30].

The cooperation between viral ligands for TLRs and gliadin has also been shown in vivo. In particular, in NOD-DQ8 mice the combination of a viral ligand, in this case polyinosinic-polycytidylic acid (Poly I:C), and dietary gliadin causes enteropathy and functional alterations of the intestinal mucosa [38].

Based on these results, the vesicular pathway regulates the innate/inflammatory response to viral ligands and bioactive dietary peptides. This suggests that together with viral infections, food proteins are able to mimic and potentiate the innate immune response to viruses and can trigger an autoimmune disease such as CD.

Gut microbiota probably has an important role in modulating the interplay between infections and gliadin to induce tissue inflammation, but their role is still not well defined.

### 2.5. Protective Role of the Mediterranean Diet in CD

**Figure 1:**
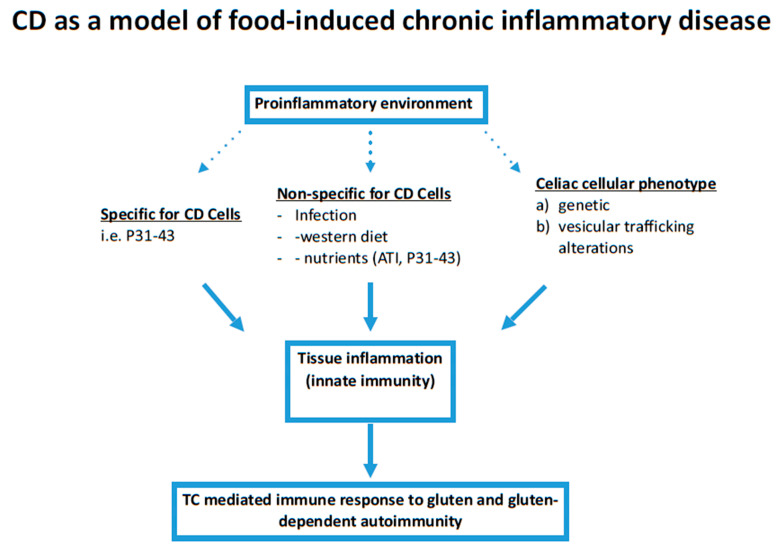
Role of the pro-inflammatory environment and tissue inflammation in CD. Pro-inflammatory environment has an important role in CD pathogenesis. It can be induced by external factors that can be specific for CD cells such as the A-gliadin peptide P31–43, or not specific (infections, diet, other wheat components etc.), but it can also be constitutive in the CD cells.

In CD, a pro-inflammatory environment exists that has different components. Gliadin and gliadin peptides can contribute and appear to be specific for CD cells, but also non-specific stimuli can contribute to this pro-inflammatory environment such as infections, a Western diet, and other wheat components such as ATIs. All this insists on a specific and yet to be completely uncovered genetic environment (Figure 1). On the other side, nutrients could also have a protective effect on CD. In this context it is interesting to note that the anti-inflammatory Mediterranean diet has a protective effect on the development of CD in children [39]. In fact, a recent prospective study by Barroso et al. was done to assess if different dietary patterns, at the age of 1 year, could be associated, or not, with the incidence of CD later on, at 6 years of age. The result of this study showed that a “prudent” Mediterranean diet (high intake of vegetables, vegetable oils, pasta and grains, and low consumption of refined cereals and sweet beverages) at 1 year of age was associated with significantly lower CD autoimmunity by 6 years of age. The protective effect of the MD on the risk of developing CD may be explained by the anti-inflammatory potential of this diet, partially mediated by an interplay with the gut microbiota [39].

It is interesting to note that the MD has an important preventive role in one other chronic inflammatory disease of the intestine, i.e., Crohn’s disease. The current literature has some new and promising data concerning the effects of the Mediterranean diet on Crohn’s disease. Greater adherence to the Mediterranean diet has been found associated with a significantly lower risk of later-onset Crohn’s disease and MedDiet score correlated with improved quality of life in a population of Crohn’s disease patients. Furthermore, evidence of a significant association between healthy lifestyle, including MD adherence, and reduced mortality in inflammatory bowel diseases (IBDs) patients has been found. Moreover, the MD seems to be most beneficial for elderly patients. Overall, the MD is considered to have a high potential to modulate gut inflammation and to be a therapeutic and preventive tool for intestinal chronic inflammatory disease [40].

## 3. Discussion

Tissue inflammation represents the “common soil” of multifactorial diseases, such as chronic inflammatory rheumatic disease or type 2 diabetes, obesity, inflammatory bowel diseases, cardiovascular and neurodegenerative diseases, asthma, cancer, and ageing [23]. These diseases are increasing and have been connected to Western lifestyle and diet [41]. This seems to be true also for CD, whose incidence has greatly increased in the last years reaching a global seroprevalence of circa 1.4 percent [42,43]. Gliadin is the antigen that causes CD when a TC-mediated immune reaction is mounted against it and an autoimmune response happens in a pro-inflammatory environment. This pro-inflammatory environment is probably due to different factors, such as infections and a Western diet, but is also due to the peculiar characteristics of wheat proteins, such as ATI and gliadin, that by themselves, with different mechanisms, can contribute to an inflammatory environment. Interestingly, combinations of pro-inflammatory events can have additive effects. Example of this are gliadin and viruses that can cooperate to induce inflammatory pathways both in vitro and in vivo (Figure 1). Though both gliadin and ATI can induce inflammation in several tissues and cells, only gliadin seems to have an effect that is more specific in CD. In this context, the undigested gliadin peptide P31–43 represents a good model of a nutrient able to induce inflammation also in normal subjects, but in a more prolonged and intense way and at lower concentrations than in CD [44].

Why does this peptide appear to be toxic to celiac patients but not to other non-celiac subjects? To answer this question, we have to look for constitutive alterations of CD cells and tissues that can render them more sensitive to gluten and other pro-inflammatory events.

Several reports note constitutive, gluten-independent alterations of CD cells. They have been studied in the normalized intestinal biopsies and organoids derived from stem cells of patients in the remission phase of the disease on a gluten-free diet and in cells obtained from tissues far away from the intestine, the primary site of inflammation [45,46]. The constitutive alterations described in the literature point to several different biological pathways such as structural cellular alterations, signaling/proliferation, stress/innate immunity, and inflammation [46]. A notable example is the NFkB pathway, constitutively altered in CD, with more than 20 components of the pathway increased in gluten-free diet (GFD-CD) biopsies [45,47,48,49,50]

A constitutive inflammatory environment in CD cells is confirmed both in skin and intestinal fibroblasts from CD patients at the remission phase of the disease with an increase of the activation of NFkB and Mitogen-Activated Protein Kinase (MAPK1) in the absence of gluten [44]. Interestingly, these cells are more sensitive to the gliadin peptide P31–43 effects both on inflammation and on innate immunity pathways. The increased sensitivity of the celiac cells to the pro-inflammatory action of gliadin is probably due to a constitutive defect in vesicular trafficking [44]. On the other side it is consolidated knowledge that innate immune and inflammatory response are linked to vesicular trafficking in several different ways [50]. A comprehensive review on P31–43 as an inducer of multiple pro-inflammatory effects can be found in Reference [26]. In conclusion, several environmental factors can contribute to the pro-inflammatory environment in CD, and a pivotal role is played by infections and food, in particular gliadin.

## 4. Materials and Methods

We have selected the most recent literature on CD, viral infections, and diet with 37/49 references from the last 10 years and 26/49 from the last 4 years.

## 5. Conclusions

The observations discussed in this commentary could have an important impact on the understanding of CD pathogenesis and also on clinical practice. The protective role of anti-rotavirus vaccination has still to be completely established, but at least theoretically the vaccine could prevent or delay the onset of the disease in children at risk [11]. It could be even reasonable to reduce the inflammatory insults during enterovirus infections by introducing a gluten-free diet for preventive purposes in subjects at genetic risk for CD. An important clinical practice to be implemented should be the introduction of the MD very early in life, at weaning, as a general way to prevent inflammation and dysbiosis.

## Figures and Tables

**Table 1:** Similarity of P31–43, viral ligands, and delay of the endocytic trafficking effects on Caco2 * cells, intestinal biopsies from CD patients ** and mice ***, on different pathways as indicated.

Pathways	HRS Silencing *	P31–43 * and **	Loxorubine *	ATIs	Poly I:C ***
**Delay of vesicular** **trafficking (EEA1, LAMP2, EGFR)**	YES	YES	YES	Not tested	Not tested
**Activation of innate** **immunity (IL15, IL15Ralpha)**	YES	YES	YES	Yes	YES
**Activation of inflammatory markers (NFkB and MAPK)**	YES	YES	YES	Yes	YES
**TLRs** **activation (MXA, Mydd 88, INF-alpha)**	YES	YES	YES	Yes	YES

## Data Availability

Not applicable.

## Abbreviations

ATI	Alpha-amylase/trypsin inhibitors
CD	Celiac Disease
GFD-CD	Gluten Free Diet-CD
HLA	Human Leukocyte Antigen
HRS	Hepatocyte growth factor-regulated tyrosine kinase substrate
MAPK	Mitogen-Activated Protein Kinase
NFkB	Nuclear factor kappa-light-chain-enhancer of activated B cells
NOD	Non-Obese-Diabetic
Poli I:C	Polyinosinic-polycytidylic acid
TC	T cells
TLR	Toll Like Receptor
